# Supplementary material for: Identification of GA2ox Family Genes and Expression Analysis under Gibberellin Treatment in Pineapple (Ananas comosus (L.) Merr.)
Source: Plants (Basel). 2023 Jul 17;12(14):2673. doi: 10.3390/plants12142673 (PMC10383957; doi:10.3390/plants12142673)
Supplement: Supplementary file 1 [file plants-12-02673-s001.zip › Table S1.pdf]

Table S1. The primers used for quantification.

Primer	Sequences (5′—3′)	Usage
Q-PCR AcActin-F	CTGGCCTACGTGGCACTTGACTT	Reference
Q-PCR AcActin-R	CACTTCTGGGCAGCGGAACCTTT	Reference
Q-PCR AcGA2ox7 F	TCACCCTCTAGTAGCGAACATA	q-PCR
Q-PCR AcGA2ox7 R	CTGACAAGGCCAGTCACTAAA	q-PCR
Q-PCR AcGA2ox41 F	TTGAGGTGATGGACGCTAAC	q-PCR
Q-PCR AcGA2ox41 R	GTGCTTTACGTTGTGCAGTATC	q-PCR
Q-PCR AcGA2ox38 F	ACGTGCTGGAGCGAATG	q-PCR
Q-PCR AcGA2ox38 R	GGGTAGTGGTTCACCCTAAA	q-PCR
Q-PCR AcGA2ox13 F	GAAGTGGAGAAACTGGCCTTTA	q-PCR
Q-PCR AcGA2ox13 R	GCTAGTTTGATCCGCGAAGA	q-PCR
Q-PCR AcGA2ox9 F	TTATCAGTAGGGCTAGGTCTCG	q-PCR
Q-PCR AcGA2ox9 R	CGGCGGGTAATAGTTGATCTT	q-PCR
Q-PCR AcGA2ox39 F	CTGGAGGGAGATTGTGACTTTC	q-PCR
Q-PCR AcGA2ox39 R	TTCTCGCTGTACGACTCCA	q-PCR
Q-PCR AcGA2ox5 F	TCCCAATCATCAACATGGAGAA	q-PCR
Q-PCR AcGA2ox5 R	CGTGATACCGTGGTTCAGAAT	q-PCR
Q-PCR AcGA2ox10 F	CCTCTCTCCAGAACTGCTACT	q-PCR
Q-PCR AcGA2ox10 R	ACACGAGGAACTGAGGAAGA	q-PCR
Q-PCR AcGA2ox15 F	AGCGCCCAGGAAATGTTAT	q-PCR
Q-PCR AcGA2ox15 R	TGCAAGTTCTTCTCCGTTAGG	q-PCR
Q-PCR AcGA2ox22 F	GTGGAGGAGAAAGCCAAGTATTA	q-PCR
Q-PCR AcGA2ox22 R	CGAATCCCGTTCTGATCATAGG	q-PCR
