# Supplementary material for: Identification of GA2ox Family Genes and Expression Analysis under Gibberellin Treatment in Pineapple (Ananas comosus (L.) Merr.)
Source: Plants (Basel). 2023 Jul 17;12(14):2673. doi: 10.3390/plants12142673 (PMC10383957; doi:10.3390/plants12142673)
Supplement: Supplementary file 1 [file plants-12-02673-s001.zip › Table S2.pdf]

Table S2. Primers used for subcellular localization.

Primer	Sequences (5′—3′)	Usage
Ubi-AcGA2ox41 F	AATTCGAGCTCGGTACCCTGCAGCTAGAAGTGAGAGGGCTT	Amplification
Ubi-AcGA2ox41 R	ccttgctcaccatCCCTGCAGCTAGAAGTGAGAGGGCTT	Amplification
Ubi-AcGA2ox38 F	AATTCGAGCTCGGTACCCATGGTGGTCTTAGCCGAGCC	Amplification
Ubi-AcGA2ox38 R	ccttgctcaccatCCCCTTCTTCTCGAACTGTCCGAGCCT	Amplification
Ubi-AcGA2ox13 F	AATTCGAGCTCGGTACCCATGGTGGAGGTGGATCCGGC	Amplification
Ubi-AcGA2ox13 R	ccttgctcaccatCCCAACAACGTTCCTGAAATGGTAAATTTG	Amplification
Ubi-AcGA2ox9 F	AATTCGAGCTCGGTACCCATGGACAAAATATTTGAGTACTTATCA	Amplification
Ubi- AcGA2ox9 R	ccttgctcaccatCCCCTGAGGGAGCTTATTAATCTTGCAAT	Amplification
Ubi-AcGA2ox39 F	AATTCGAGCTCGGTACCCATGCTGCTGCAGGGAGAAGCA	Amplification
Ubi-AcGA2ox39 R	ccttgctcaccatCCCAGCCAAGATTTCATTGAGAGATTTGGG	Amplification
Ubi-AcGA2ox5 F	AATTCGAGCTCGGTACCCATGGAGAGTAAGTTCCCAATCATCAAC	Amplification
Ubi-AcGA2ox5 R	ccttgctcaccatCCCCTGGGTTGCGATCGGCGGC	Amplification
Ubi-AcGA2ox10 F	AATTCGAGCTCGGTACCCATGGCTGCTGAAGCTTTCACC	Amplification
Ubi AcGA2ox10 R	ccttgctcaccatCCCCTCTCTTGCATTCAATCTAAGAAGATCTA	Amplification
Ubi AcGA2ox15 F	AATTCGAGCTCGGTACCCATGGCGAAGCTTCTCTCCGAT	Amplification
Ubi-AcGA2ox15 R	ccttgctcaccatCCCGCTAGTTTTATTCTTAAAGCCTTCCGC	Amplification
Ubi-AcGA2ox22 F	AATTCGAGCTCGGTACCCATGGAGCTCCTCTCCAGCGG	Amplification
Ubi-AcGA2ox22 R	ccttgctcaccatCCCCTCTTCAATCTTGAAGTACTCCAACAC	Amplification
Ubi-AcGA2ox7 F	AATTCGAGCTCGGTACCCATGGTTGTGATCCCGGTGGTG	Amplification
Ubi-AcGA2ox7 R	ccttgctcaccatCCCTGCAGCTAGAAGTGAGAGGGCT	Amplification
