# Supplementary figures and images for: Identification of GA2ox Family Genes and Expression Analysis under Gibberellin Treatment in Pineapple (Ananas comosus (L.) Merr.)
Source: Plants (Basel). 2023 Jul 17;12(14):2673. doi: 10.3390/plants12142673 (PMC10383957; doi:10.3390/plants12142673)

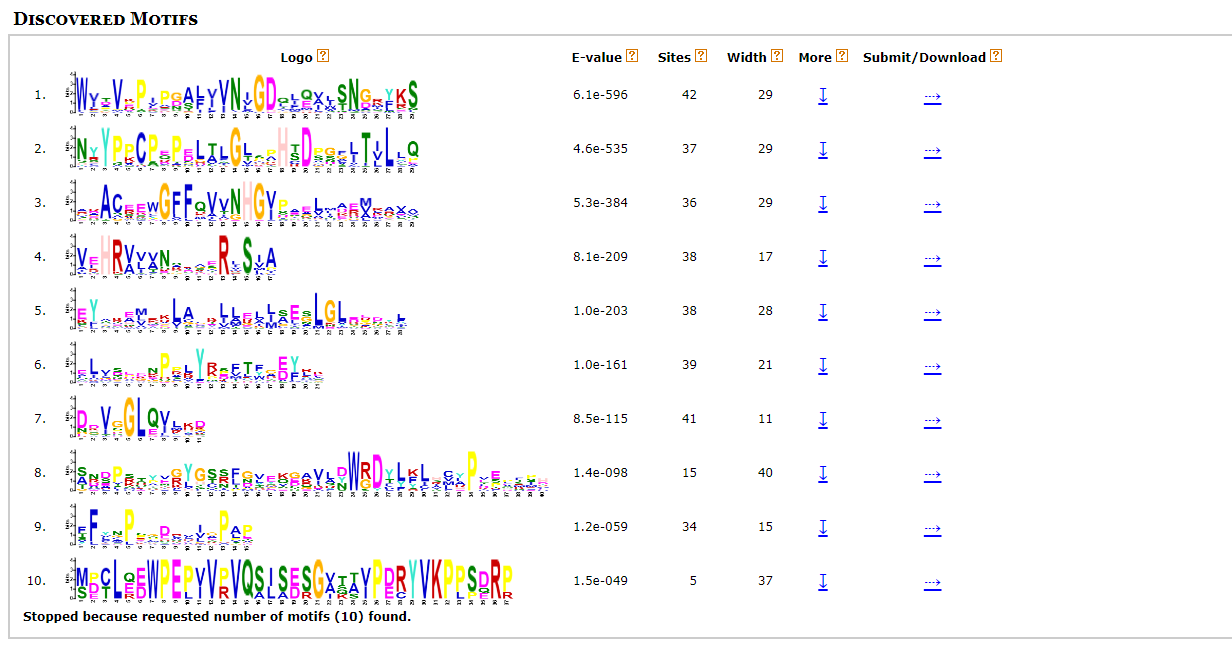

Supplement: Supplementary file 1 [file plants-12-02673-s001.zip › Figure S1/10 motifs.png]

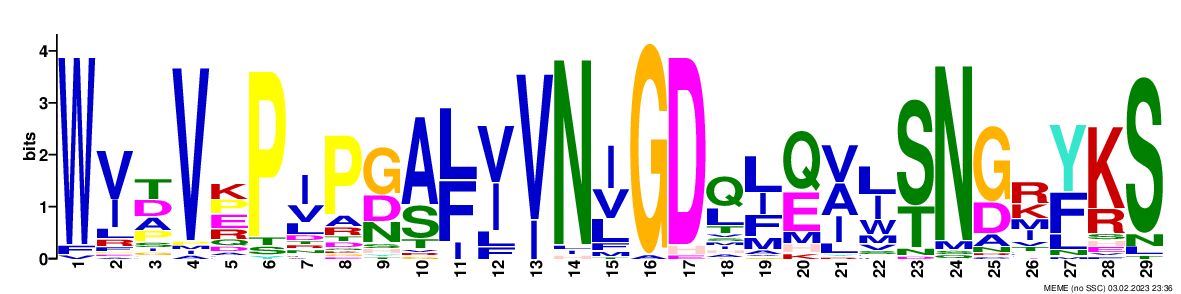

Supplement: Supplementary file 1 [file plants-12-02673-s001.zip › Figure S1/motif1.png]

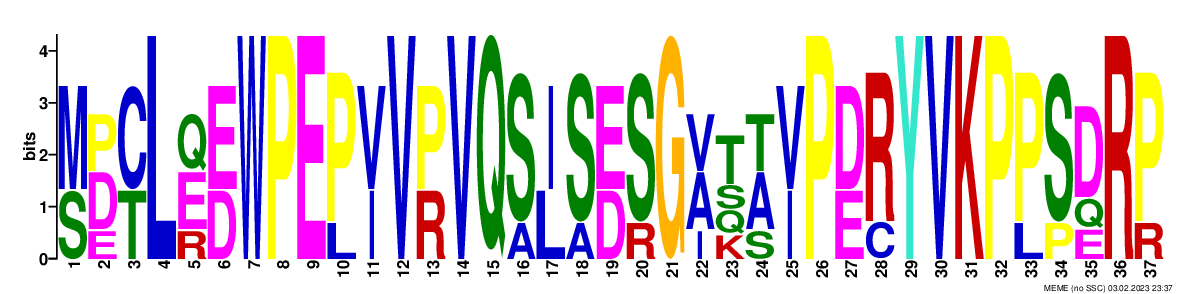

Supplement: Supplementary file 1 [file plants-12-02673-s001.zip › Figure S1/motif10.png]

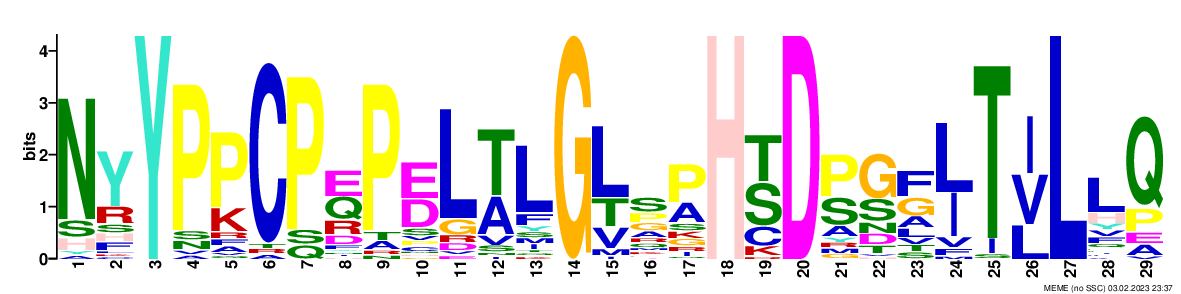

Supplement: Supplementary file 1 [file plants-12-02673-s001.zip › Figure S1/motif2.png]

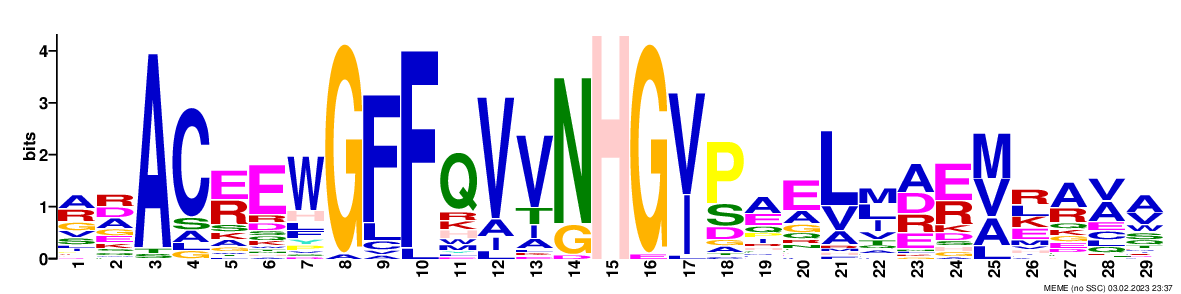

Supplement: Supplementary file 1 [file plants-12-02673-s001.zip › Figure S1/motif3.png]

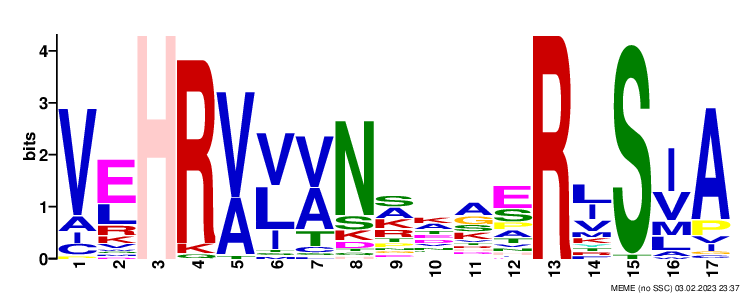

Supplement: Supplementary file 1 [file plants-12-02673-s001.zip › Figure S1/motif4.png]

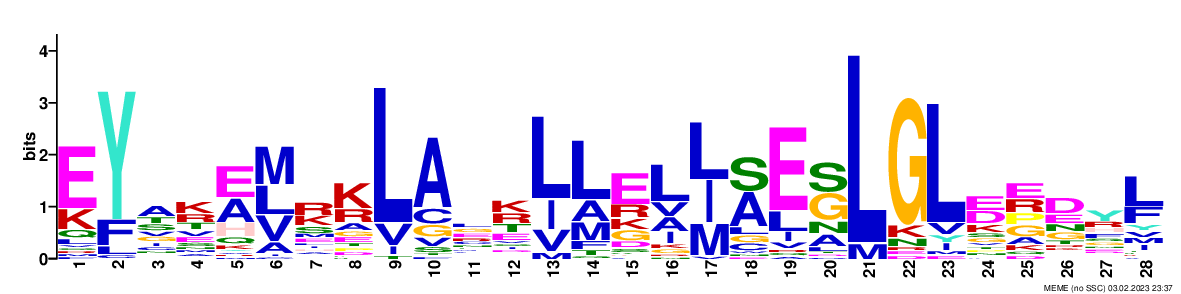

Supplement: Supplementary file 1 [file plants-12-02673-s001.zip › Figure S1/motif5.png]

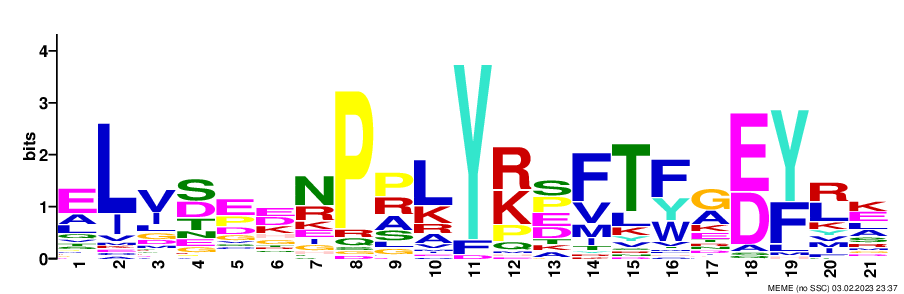

Supplement: Supplementary file 1 [file plants-12-02673-s001.zip › Figure S1/motif6.png]

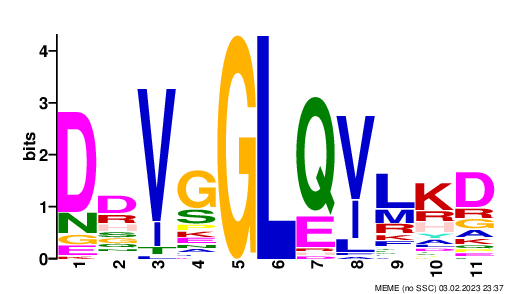

Supplement: Supplementary file 1 [file plants-12-02673-s001.zip › Figure S1/motif7.png]

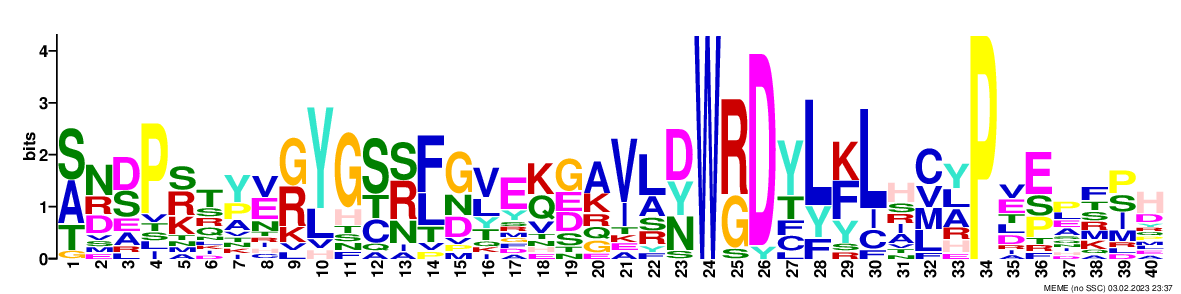

Supplement: Supplementary file 1 [file plants-12-02673-s001.zip › Figure S1/motif8.png]

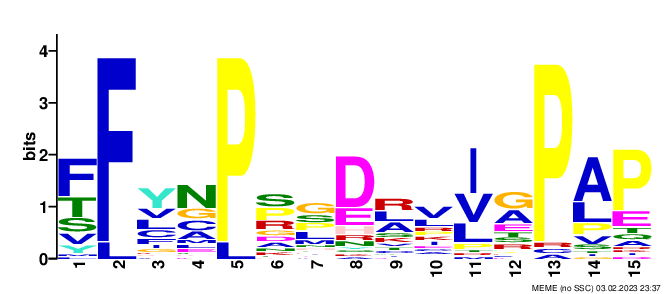

Supplement: Supplementary file 1 [file plants-12-02673-s001.zip › Figure S1/motif9.png]
